# *In vivo* molecular imaging of neuroinflammation in Alzheimer's disease

**DOI:** 10.1111/jnc.14615

**Published:** 2018-11-26

**Authors:** Aisling Chaney, Steve R. Williams, Herve Boutin

**Affiliations:** ^1^ School of Health Sciences Division of Informatics Faculty of Biology, Medicine and Health Manchester Academic Health Sciences Centre University of Manchester Manchester UK; ^2^ Wolfson Molecular Imaging Centre Faculty of Biology, Medicine and Health and Manchester Academic Health Sciences Centre University of Manchester Manchester UK; ^3^ School of Biological Sciences Division of Neuroscience and Experimental Psychology Faculty of Biology, Medicine and Health Manchester Academic Health Sciences Centre University of Manchester Manchester UK; ^4^ Present address: 1201 Welch Road, P‐255, Stanford University Palo Alto CA 94305 USA.

**Keywords:** Alzheimer's disease, magnetic resonance spectroscopy, neuroimaging, neuroinflammation, positron emission tomography, TSPO

## Abstract

It has become increasingly evident that neuroinflammation plays a critical role in the pathophysiology of Alzheimer's disease (AD) and other neurodegenerative disorders. Increased glial cell activation is consistently reported in both rodent models of AD and in AD patients. Moreover, recent genome wide association studies have revealed multiple genes associated with inflammation and immunity are significantly associated with an increased risk of AD development (e.g. TREM2). Non‐invasive *in vivo* detection and tracking of neuroinflammation is necessary to enhance our understanding of the contribution of neuroinflammation to the initiation and progression of AD. Importantly, accurate methods of quantifying neuroinflammation may aid early diagnosis and serve as an output for therapeutic monitoring and disease management. This review details current *in vivo* imaging biomarkers of neuroinflammation being explored and summarizes both pre‐clinical and clinical results from molecular imaging studies investigating the role of neuroinflammation in AD, with a focus on positron emission tomography and magnetic resonance spectroscopy (MRS).

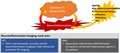

**Abbreviations used**
AD: Alzheimer's disease
APP: amyloid precursor protein
Aβ: amyloid beta
BP: binding potential
CB2: cannabinoid type 2 receptor
CDR: clinical dementia rating
Cho: choline‐containing compounds
CNS: central nervous system
Cre: creatine & phosphocreatine
DED: deuterium‐L‐deprenyl
HAB: high‐affinity binders
HC: healthy controls
IL‐1: interleukin‐1
LAB: low‐affinity binders
LPS: lipopolysaccharide
MAB: mixed‐affinity binders
MCI: mild cognitive impairment
mI: myo‐Inositol
MMSE: mini–mental state examination
MRS: magnetic resonance spectroscopy
NAA: N‐acetylaspartate
NFT: neurofibrillary tangles
NMR: Nuclear magnetic resonance
PBBS: peripheral benzodiazepine binding site
PBR: peripheral‐type benzodiazepine receptor
PET: positron emission tomography
PS1: presenilin‐1
PSP: progressive supranuclear palsy
ScyI: scyllo‐Inositol
TG: transgenic
TNF‐α: tumour necrosis factor‐α
TSPO: translocator protein 18 kDa
WT: wild type

Alzheimer's disease (AD), is the most common form of dementia, a group of debilitating and progressive neurodegenerative disorders characterised by the deterioration of cognitive, intellectual and emotional function (Prince *et al*. 2013; Lambert *et al*. 2014). Dementia has become a major international health problem affecting approximately 46.8 million people worldwide and costing a staggering US$818 billion a year (Prince *et al*. 2016; Scheltens *et al*. 2016). With the biggest risk factor for AD being age, and with generations continuing to grow older, the incidence of dementia worldwide is estimated to rise to over 131 million people by 2050 (Prince *et al*. 2016), causing a huge impact on families, society and the economy.

Pathologically, AD is characterised by extracellular amyloid beta (Aβ) plaques and intracellular hyper‐phosphorylated Tau neurofibrillary tangles. However, the causative role of these hallmarks in disease development remains uncertain, and it is clear that many other factors are involved in AD manifestation and progression. In particular, it is increasingly recognised that neuroinflammation plays an important role in the pathophysiology of AD, whether as a cause or consequence remains unclear. However, altogether changes in neuroinflammatory processes, such as increased microglial activation and cytokine expression, observed *in vivo* (Cagnin *et al*. 2001; Edison *et al*. 2008; Nuzzo *et al*. 2014) and post‐mortem (Hayes *et al*. 2002) in AD patients as well as recent genome‐wide association studies (Neumann and Daly 2013) point towards a causative role of neuroinflammation in AD. However, with evidence of both beneficial and detrimental effects, the precise contribution of neuroinflammation to AD remains to be determined.

As a result of the idiopathic nature of AD, current treatments are restricted to symptomatic therapy and do not cure, prevent nor halt disease progression. Consequently, a huge unmet need exists to unravel the mechanisms underlying AD and find robust non‐invasive biomarkers to aid the diagnosis and earlier detection of AD, but also the development and assessment of new therapeutic strategies. Imaging provides a safe and non‐invasive method to obtain physiologically relevant information *in vivo* about pathophysiological mechanisms, and proves an indispensable tool at both a pre‐clinical and clinical level. Here, we review how positron emission tomography (PET) and magnetic resonance spectroscopy (MRS) have contributed to our knowledge of AD, and have been used to help us understand the role of neuroinflammation and neurodegeneration in AD from the perspective of both pre‐clinical and clinical studies.

## Neuroinflammation in AD

Microglia, the resident immune cells of the CNS, play a fundamental role in brain surveillance and homeostasis (Nimmerjahn *et al*. 2005). Under physiological conditions microglia are neuroprotective, playing a key role in phagocytosis and neurotrophin release, their function is implicit to maintaining a healthy brain environment. However, in response to disease or injury microglia become activated, leading to the production and release of inflammatory cytokines, including interleukin‐1α (IL‐1α), interleukin‐1β (IL‐1β) and tumour necrosis factor‐α or reactive oxygen and nitrogen species resulting in a pro‐inflammatory response. Activated microglia also synthesise proteolytic enzymes such as Cathepsin B damaging the extracellular matrix and causing neuronal dysfunction (Wood 1998). Inflammation usually resolves itself and is essential to the repair process. However, if inflammation is prolonged, pathologic (i.e. chronic) inflammation can occur resulting in detrimental effects on brain function due to excessive or persistent release of cytotoxic factors. This persistent over‐activation of pro‐inflammatory responses has been implicated in many neurodegenerative disorders including AD.

The presence of activated microglia surrounding amyloid plaques (Haga *et al*. 1989) and increased levels of pro‐inflammatory cytokines in both the periphery and CNS (Swardfager *et al*. 2010; Rubio‐Perez and Morillas‐Ruiz 2012; Varnum and Ikezu 2012), support the key role of inflammation in AD manifestation. Forlenza *et al*. (2009) found that the serum levels of IL‐1α and IL‐1β are not only increased in AD but also in mild cognitive impairment (MCI) patients, implicating neuroinflammation as an early player in disease development. It has been reported that microglia can become ‘primed’ (prone to respond) with age, supporting the concept that, with increasing age and exposure to environmental factors the brain becomes more susceptible to mounting a stronger maladapted pro‐inflammatory response (Norden and Godbout 2013). Furthermore, AD patients deteriorate faster following infections (Nee and Lippa 1999; Holmes *et al*. 2003; Perry *et al*. 2003), which are known to induce neuroinflammation (Perry *et al*. 2003, 2007), suggesting that inflammation also has a function in disease progression.

Much work has been done pre‐clinically to investigate the role of inflammation in AD. Kitazawa *et al*. (2011) found that experimentally blocking IL‐1 receptors improved cognitive test performance in a triple transgenic (3 × Tg) mouse model of AD displaying both Aβ and tau neuropathology, suggesting that IL‐1 could be a major contributor to cognitive deficits seen in AD. Furthermore, mice with increased levels of IL‐1β and S100B (cytokine released predominantly by astrocytes) have shown exacerbated damage in response to ICV injection of human Aβ_1‐42_ (Craft *et al*. 2006), suggesting that baseline neuroinflammation may also play a role in AD development. In parallel to human findings, peripheral inflammation such as lipopolysaccharide (LPS) injection (Kitazawa *et al*. 2005; Lee *et al*. 2008), PolyI:C injection (Krstic *et al*. 2012) or pulmonary infection (McManus *et al*. 2014) has been shown to worsen neuroinflammation, pathology and cognitive performance in mouse models of disease.

Traditionally, microglia were assumed to be either in a pro‐ or anti‐inflammatory state, however recent evidence suggests that their activation is not as simplistic. Microglial activation is complex and triggered by multiple environmental stimuli, with persuasive evidence indicating a continuum of functional states (Town *et al*. 2005; Gomez‐Nicola and Perry 2015). Moreover, astrogliosis is also commonly observed in AD (Olabarria *et al*. 2010; Rodriguez‐Vieitez *et al*. 2015; Verkhratsky *et al*. 2016) and crosstalk between microglia and astrocytes leads to the generation of pro‐inflammatory/neurotoxic astrocytes (Liddelow *et al*. 2017). Whether microglial cells and astrocytes are a contributing factor to disease manifestation in AD, and when and why they switch from being neuroprotective to detrimental remains to be fully determined and is likely affected by the inflammatory burden occurring over the life‐span of patients and the course of disease. Nonetheless, as neuroinflammation is increasingly implicated in neurodegeneration, an urgent need exists to find new innovative ways of investigating its function in health and disease. Advances in medical imaging can be exploited to help us better understand the evolution of neuroinflammation at different disease states and hence elucidate underlying pathophysiological mechanisms and targets for therapeutic intervention.

## Imaging biomarkers of neuroinflammation

### Translocator protein 18 kDa (TSPO)

The translocator protein (TSPO), formerly known as the peripheral‐type benzodiazepine receptor (PBR) or peripheral benzodiazepine binding site, is an 18 kDa protein located on the outer membrane of mitochondria. TSPO is notably involved in the synthesis of neurosteroids, playing a role in cholesterol transport across the mitochondrial membrane (Brown and Papadopoulos 2001) but is also linked to neuronal survival and neuroinflammation, and has been associated with the pathology of many neurological disorders including AD (Papadopoulos *et al*. 2006). TSPO is highly expressed in the periphery, but is low in a healthy brain and restricted mainly to microglia (Scarf and Kassiou 2011) and endothelial cells (Tomasi *et al*. 2008). Numerous experimental (Imaizumi *et al*. 2007; Chauveau *et al*. 2011; Boutin and Pinborg 2015; Serriere *et al*. 2015; Thomas *et al*. 2016; Sridharan *et al*. 2017) and clinical (Gulyas *et al*. 2009; Su *et al*. 2013) studies have now demonstrated that TSPO levels directly proportional to activation of microglia and potentially of astrocytes, either through up‐regulation of TSPO by activated cells and/or increased density of activated cells and/or infiltrated macrophages (Cosenza‐Nashat *et al*. 2009; Owen *et al*. 2017). Upon microglial activation, TSPO may undergo polymerization leading to the formation of possible multiple binding sites (Scarf and Kassiou 2011). Further investigation needs to be put into understanding the significance of its multiple binding sites, as their functions remain unclear and represent a major problem preventing therapeutic and diagnostic exploitation. However, TSPO remains a promising target representing a modulatory system that links neurotransmitter dysfunction, excitotoxicity and inflammation, all of which appear to be crucial processes in AD pathophysiology.

Overall, the specific presence of TSPO on activated microglia can be exploited to image neuroinflammation *in vivo*. Radiotracers have been developed that allow the introduction of radionuclides onto TSPO ligands. As levels are low in healthy brains but appear to be elevated in disease, TSPO expression remains so far the biomarker of choice to measure non‐invasively neuroinflammation using PET.

#### Clinical TSPO PET imaging

PK11195, a potent TSPO antagonist, has been ^11^C‐labelled for use in PET imaging and demonstrated good correlation with the presence of activated microglial/macrophage in human AD tissue (Benavides *et al*. 1988; Banati 2002; Venneti *et al*. 2008). In AD, one of the first studies (Edison *et al*. 2008) investigated [^11^C]‐R‐PK11195 uptake in 13 AD patients and observed increased uptake in areas associated with AD pathology including frontal, occipital, temporal and parietal regions as well as the anterior and posterior cingulate and whole cortex compared to healthy controls. However, no significant increases in [^11^C]‐R‐PK11195 uptake where identified in the hippocampus. Amyloid imaging was also performed in these subjects using 11C‐labelled Pittsburgh compound ([^11^C]PiB), a radioactive analog of the commonly used dye thioflavin T, which binds to insoluble Aβ plaques (Agdeppa *et al*. 2001). [^11^C]PiB uptake increased in AD patients in all areas including the hippocampus and thalamus. A significant inverse correlation was observed between [^11^C]‐R‐PK11195 uptake and mini–mental state examination (MMSE) scores, but not between [^11^C]PiB and MMSE scores, suggesting that neuroinflammation, but not amyloid load, was a suitable biomarker of disease severity (Edison *et al*. 2008). Similarly, Yokokura *et al*. (2011) found that [^11^C]‐R‐PK11195 binding was significantly increased in AD patients in medial frontal, parietal, and left temporal cortex. A negative correlation was also identified with MMSE scores and [^11^C]‐R‐PK11195 uptake in left regions of the brain including the hippocampus, anterior cingulate, precuneus and medial frontal cortex. In line with Edison *et al*. (2008), no correlations were identified between [^11^C]PiB and [^11^C]‐R‐PK11195 uptake. In contrast, later studies reported no significant differences in [^11^C]‐R‐PK11195 uptake in either AD or MCI patients compared to control subjects. Schuitemaker *et al*. (2013) did not observe differences in [^11^C]‐R‐PK11195 uptake between AD or amnesic MCI patients and healthy subjects using an ROI approach. Furthermore, no correlation was found between cognitive scores and [^11^C]‐R‐PK11195 uptake. Although, small clusters of increased binding were observed within the parietal lobe of AD patients but not in prodromal or healthy participants, suggesting that inflammation in AD may be a highly‐reserved process associated with subtle focal increases. Wiley *et al*. (2009) reported a two‐fold increase in PiB retention in AD compared to control subjects in regions associated with amyloid pathology, yet no differences in [^11^C]‐R‐PK11195 retention were observed between any diagnostic groups or within PiB positive or negative groups, and no increase in [^11^C]‐R‐PK11195 uptake was found in amyloid rich areas. MCI also appears to be difficult to distinguish from healthy controls, with reports of increased (Okello *et al*. 2009) and unchanged [^11^C]‐R‐PK11195 uptake (Wiley *et al*. 2009). A review by Hommet *et al*. (2014) evaluated six studies investigating the link between neuroinflammation and amyloid load by either [^11^C]‐R‐PK11195 or [^11^C] C‐deuterium‐L‐deprenyl (DED) (which binds to monoamine oxidase B in astrocytes) and PiB PET in AD and came to the same conclusions. No correlation was reported between [^11^C]‐R‐PK11195 and PiB, however a correlation was found between [^11^C]DED and PiB, suggesting that astrocyte activation may be directly related to plaque load but microglial activation may work independently via a different mechanism such as through soluble Aβ or tau. In contrast, a more recent follow‐up study by Fan *et al*. (2015) investigating [^11^C]‐R‐PK11195, [^11^C]PiB, and [^18^F]FDG uptake as well as correlations between these at a voxel level in AD patients and healthy controls showed that [^11^C]‐R‐PK11195 uptake was positively correlated with amyloid load and negatively correlated with glucose metabolism in the AD cohort. In addition, the regions with increased [^11^C]‐R‐PK11195 uptake at baseline differed from the regions affected upon follow up, which is line with the theory that neuroinflammation can change with disease progression. On the other hand, a recent study suggested that neuroinflammation plays a critical role in the early neurodegenerative process in amyloid positive MCI cohorts. Parbo *et al*. (2017) identified increased neuroinflammation using [^11^C]‐R‐PK11195 in a high majority (85%) of amyloid positive (PiB‐positive) MCI patients – the clinical cohort that is at the highest risk of progression to AD. This study revealed multiple cortical clusters in PiB‐positive MCI subjects with elevated microglial activation compared to PiB‐negative MCI and HCs. Additionally, a positive correlation between PiB and [^11^C]‐R‐PK11195 was observed in clusters located in the frontal, parietal and temporal cortices. However, 15% of PiB‐positive MCI subjects displayed [^11^C]‐R‐PK11195 uptake within the normal range, and 25% of PiB‐negative MCI subjects displayed increased [^11^C]‐R‐PK11195 binding, indicating that it is possible to have elevated neuroinflammation without amyloid burden and vice versa. Moreover, [^11^C]‐R‐PK11195 is also able to highlight different anatomical patterns in AD vs other type of related dementia such as progressive supranuclear palsy(PSP)‐Richardson syndrome as recently demonstrated by Passamonti *et al*. (2018). This study showed that both AD and PSP patients had elevated [^11^C]‐R‐PK11195 when compared to controls but that increased neuroinflammation was distinctively increased in medial temporal and occipital, temporal, and parietal cortices in AD whereas PSP patients had increased neuroinflammation in thalamus, putamen, and pallidum. Interestingly, in both pathologies, increased neuroinflammation was correlated with disease severity (in cuneus/precuneus for AD and in pallidum, midbrain, and pons for PSP) (Passamonti *et al*. 2018). Overall from all the studies using [^11^C]‐R‐PK11195 in AD, it emerges that there are overlaps between AD and HC populations and that measuring relatively modest changes in TSPO levels (10‐30%) in neurodegenerative diseases is still challenging. The overlap between controls and AD patients obtained with [^11^C]‐R‐PK11195 might be due to its high non‐specific binding, resulting in a poor signal‐to‐noise ratio (Boutin and Pinborg 2015; Varrone and Lammertsma 2015), which certainly limits the sensitivity of the measure. Another parameter that may have contributed to the discrepancy between the studies of Schuitemaker *et al*. (2013), Wiley *et al*. (2009) and all the others is the difficulty to model [^11^C]PK11195 binding. All the studies, apart from Yokokura *et al*. (2011) who used an input reference curve from the control population, chose the supervised cluster analysis (Lammertsma and Hume 1996; Kropholler *et al*. 2005, 2006) to determine the reference tissue used to calculate the binding potential (BP), Wiley *et al*. (2009) used the cerebellum as the reference region. It is interesting to note that all studies, using cluster analysis except for Schuitemaker *et al*. (2013) reported significant differences between controls and AD whereas Wiley *et al*. (2009) did not find any significant increase in [^11^C]PK11195 uptake. So overall, there is some consistency in showing an increase in neuroinflammation in AD, using [^11^C]PK11195, but the method used to quantify the TSPO binding should be carefully considered (Kropholler *et al*. 2007) and the *supervised* cluster analysis (Turkheimer *et al*. 2007) being the favoured method. This observation triggered the search for and the development of more sensitive TSPO tracers over the past two decades. Much effort has been put into this and 41 second/third generation tracers have been developed (Chauveau *et al*. 2008; Liu *et al*. 2014) that possess higher affinities than [^11^C]‐R‐PK11195 such as [^18^F]‐FEPPA (Suridjan *et al*. 2014), [^11^C]DAA1106 (Fujimura *et al*. 2006), [^11^C]DPA‐713 (Boutin *et al*. 2007), [^18^F]DPA‐714 (Chauveau *et al*. 2009; Doorduin *et al*. 2009; Boutin *et al*. 2013), [^18^F]GE‐180 (Dickens *et al*. 2014; Boutin *et al*. 2015) and [^11^C]PBR28 (Imaizumi *et al*. 2007; Fujita *et al*. 2008). However, the identification of two binding sites on TSPO, initially with [^11^C]PBR28 (Fujita *et al*. 2008) but that affects all second/third‐generation tracers to some extent, has initially slowed down a more generic clinical use of these new TSPO radio‐ligands. It was later discovered that these differences in binding characteristics were due to the TSPO polymorphism (rs6971) (Owen *et al*. 2012), leading to the TSPO‐binding site being either of high or low affinity (Owen *et al*. 2010), with subjects displaying high, low or mixed affinities (both high and low affinity alleles). This discovery has led to the generic use of genotyping for the polymorphism rs6971 to identify high, low and mixed‐affinity binders [high‐affinity binders (HAB), low‐affinity binders (LAB) and mixed‐affinity binders (MAB)] and to a faster implementation of these new TSPO tracers in clinical studies. The potential advantages of new TSPO tracers has been recently illustrated by the study of Yokokura *et al*. (2017) comparing [^11^C]DPA‐713 and [^11^C]‐R‐PK11195 in the same subjects. This study shows that, as supported by the initial preclinical studies, [^11^C]DPA‐713 has a higher capacity to detect subtle increases in TSPO levels than [^11^C]‐R‐PK11195. Importantly, significant differences were not only identified in AD patients in various brain regions but also in normal aging (young vs. elderly controls); finally more brain regions were detected having increased neuroinflammation, using [^11^C]DPA‐713 than with [^11^C]‐R‐PK11195. Without comparing other new tracers to [^11^C]‐R‐PK11195, earlier studies had already taken advantages of various new TSPO tracer. Kreisl *et al*. (2013) investigated TSPO binding in AD, MCI and control subjects, using [^11^C]PBR28 and observed a significant increase in uptake in AD patients compared to MCI and control subjects in cortical regions, including prefrontal, inferior parietal, superior, medial and inferior temporal, and occipital regions. When subjects were stratified by genotype into HAB and MAB, uptake was increased in the parietal cortex in HAB AD patients when compared to both MCI and healthy subjects. However, MAB AD patients had a significant increase in uptake when compared to healthy subjects but only a trend toward significance was reached when compared to MCI patients. No differences were found between MCI and healthy subjects, suggesting that increased inflammation is a specific characteristic in the progression from MCI to AD. However, it must be noted that [^11^C]PBR28 has the highest differential (~40 fold) in affinity between the low and high affinity binding sites; it is therefore not suitable to image low affinity binders and will have poor sensitivity in imaging mixed‐affinity binders due to the large reduction in binding due to 50% of the binding sites being low affinity. Moreover the pharmacokinetic characteristics of [^11^C]PBR28 seem to make it particularly challenging to model (Rizzo *et al*. 2017). Despite this, the same group has recently published a 2.7 years follow‐up study showing that (i) AD patients had a 4–6% increase in TSPO binding per annum whereas it was only 0.5–1% in HC and (ii) AD patients with the highest level and increase in TSPO binding had faster brain atrophy and cognitive decline (Kreisl *et al*. 2016). Similarly, Yasuno *et al*. (2012) investigated binding in AD, MCI and healthy subjects with a 5 year follow‐up using [^11^C]DAA1106. Mean BP values were significantly increased in AD and MCI patients when compared to controls in brain regions including prefrontal, parietal, occipital and cingulate cortices, striatum, and thalamus; however no significant differences in BPs were identified between MCI and AD patients. No correlation was found between psychological scores and BP values in any region. The 5‐year follow up revealed that all the MCI patients that developed dementia had significantly increased [^11^C]DAA1106 uptake compared to controls, suggesting again that progression from MCI to AD is linked to an increased neuroinflammation. A more recent study using [^18^F]DPA‐714 in AD patients, prodromal AD and healthy controls confirmed that this second‐generation TSPO tracer can detect increased inflammation in the AD brain (Hamelin *et al*. 2016). This study showed that all AD patients, but most predominantly the prodromal AD cohort, demonstrated elevated [^18^F]DPA‐714 uptake in the temporo‐parietal cortex when compared to controls regardless of their TSPO genotype. This study also demonstrated that [^11^C]PiB positive controls displayed higher [^18^F]DPA‐714 binding than controls. Moreover, TSPO levels were correlated with cognitive score, gray matter volumes and PiB binding. Interestingly, Hamelin *et al*. (2016) found that slow decliner (clinical dementia rating score assessed over a 2‐year period) had higher [^18^F]DPA‐714 binding than fast decliner despite comparable amyloid levels, suggesting that higher level of neuroinflammation might be protective. Taken altogether, the studies by Kreisl *et al*. (2016), Yasuno *et al*. (2012) and Hamelin *et al*. (2016) indicates that more than the level of neuroinflammation at a given, punctual, stage of the disease, the time‐course and progression of neuroinflammation is the most important parameter, supporting the need for longitudinal studies in AD cohorts. Overall, these studies clearly demonstrate a role for neuroinflammation in AD and the possible use of TSPO imaging as biomarker of disease progression. These studies however also raise important questions regarding the time‐course and role of neuroinflammation as well as the biological meaning of TSPO expression, the potential multiple binding sites possibly affecting differentially the binding of various TSPO tracers, and the use of TSPO imaging as a prognosis/therapy outcome measure in AD. Further work is still needed to truly understand the meaning of TSPO expression in terms of glial phenotype and biology to elucidate the detrimental from the beneficial effects of gliosis. Furthermore, there are contradictory results regarding the correlations (or lack of) between increased TSPO binding, as found in the majority of studies, and increased PiB uptake and/or cognitive deficit. This suggests that neuroinflammation and Aβ plaque load may have different pathological time‐courses, which depending on the time‐point of observation may be correlated or not. These data also suggest that neuroinflammation may have an early role to play in the disease. Development of new biomarkers and tracers for neuroinflammation and correlation of neuroinflammation readouts with better tau and soluble amyloid tracers as well as more follow‐up studies should help to resolve these interrogations.

#### Pre‐clinical PET imaging

Historically, *in vivo* TSPO PET imaging was first performed in patients as clinical PET scanners were available long before dedicated pre‐clinical PET scanners. Nevertheless, over the past decades, TSPO pre‐clinical imaging in rodent models of neurodegeneration has allowed the development of the second generation of TSPO tracers and their validation and investigational uses in disease models, including AD models. A study by Rapic *et al*. (2013) showed [^11^C]‐R‐PK11195 uptake to be increased (~+23%) in the whole brain of 15‐month‐ old amyloid precursor protein APP_swe_×PS1_Δe9_ mice compared to WT; however, this did not remain significant after multiple comparisons. Although increased microglia activation around amyloid plaques was evident in the transgenic (TG) mice by 12.5 months, no difference in [^11^C]‐R‐PK11195 uptake was evident at 13 months of age. This study suggests that [^11^C]‐R‐PK11195 is not sensitive enough to pick up early microglial changes in this mouse model of AD. In contrast, Venneti *et al*. (2009) found increased [^11^C]‐R‐PK11195 binding in the same mouse model at 16‐19 months but not at the earlier age of 13–16 months. This coincided with increases in Iba‐1 staining at 16–19 months but not at 13–16 months, suggesting that [^11^C]‐R‐PK11195 can detect increases in microglial activation in contrast to Rapic *et al*. However, this could be at least partly explained by the larger age gap in the time‐points.

The TSPO polymorphism does not occur in animals and therefore new tracers with improved kinetics and affinities can be used pre‐clinically to investigate microglial activation without this particular issue. Pre‐clinical use of PET has many advantages over its use in a clinical research setting including confirmation of results by *ex vivo* means and correlation with other *ex vivo* modalities (e.g. histology, autoradiography). This can be exploited to explore the relationship between AD pathology and neuroinflammation. Increased [^18^F]GE‐180 uptake was found in the hippocampus of 26 month old APP_swe_×PS1_Δe9_ mice compared to wild‐type (WT) mice and young 4‐month‐old TG mice, demonstrating that both normal aging and disease have an effect on microglial activation (Liu *et al*. 2015). These results were supported by increased [^18^F]GE‐180 binding in *ex vivo* autoradiography and increased TSPO immunoreactivity, reinforcing its use as a viable TSPO tracer *in vivo*. Similarly, increased uptake of an alternative TSPO tracer, [^18^F]PBR06, was observed *in vivo* in the cortex and hippocampus of APP^L/S^ mice when compared to WT mice at 16 months of age, which was supported by *ex vivo* autoradiography and CD68 staining (James *et al*. 2015). However, *ex vivo* analysis revealed that there was also an increase in [^18^F]PBR06 uptake and CD68 staining at 9–10 months that was not detected, using PET analysis *in vivo*. These results indicate that either [^18^F]PBR06 was not sensitive enough as a tracer or TSPO PET as a technique to pick up subtle neuroinflammatory changes earlier in disease. More recently, we also demonstrated that [^18^F]DPA‐714 uptake was increased significantly at 18 months of age in APP_swe_×PS1_Δe9_ but not at earlier time‐points (6 and 12 months) (Chaney *et al*. 2018). A trend to increase with age was also observed in WT animals, reducing the difference between TG and WT at 12 and 18 months of age, confirming that neuroinflammation increases with age and making detection of pathological neuroinflammation more difficult to detect in aging populations. However, the presence of neuroinflammation (activated astrocytes and microglia around Aβ plaques) was confirmed by immunohistochemistry particularly at 12 months of age, once again pointing toward a lack of sensitivity of *in vivo* PET in this mouse model (Chaney *et al*. 2018). It must be noted that investigations in rodents are limited by the spatial resolution of the scanner when compared to the small size of the mouse brain, a general limitation associated with all macroscopic (i.e. PET, SPECT) pre‐clinical modalities. Hence, autoradiography has also been used to investigate the relationship between neuroinflammation and amyloid or tau pathology. Ji *et al*. (2008) looked at [^18^F]FEDAA1106 uptake in brain slices of an amyloid (APP23 mouse) and tau (PS19 mouse) pathology model of AD. Increased [^18^F]FEDAA1106 uptake was found in the hippocampus and entorhinal cortex of the amyloid model at 20 months and the tau model at 9 months of age. A co‐localisation of GFAP (glial fibrillary acidic protein; astrocyte marker) and TSPO staining in close proximity to both Aβ pathology in the APP23 mouse was observed, however CD11b (cluster of differentiation molecule 11B; microglial marker) positive cells displayed very low levels of TSPO expression. The opposite was seen in the PS19 tau model, with TSPO co‐localising well with CD11b positive cells but not with GFAP positive cells, suggesting that tau and amyloid pathology may alter neuroinflammation status differently via activation of different glial cell populations. Increased [^3H^]DAA1106 was also found *ex vivo* in the hippocampus and cortex of the P301S tau model as early as 3 months of age and prior to tau pathology becoming evident in this model (Yoshiyama *et al*. 2007), reinforcing the hypothesis of an early role of neuroinflammation in both tau and amyloid pathology manifestation.

Multiple‐tracer studies have been performed pre‐clinically to investigate the relationship between neuroinflammation and other characteristics of disease. Serriere *et al*. (2015) investigated amyloid and pathology and neuroinflammatory status in the APP_swe_×PS1_Δe9_ mouse model using [^18^F]DPA‐714 and [^18^F]AV‐45 (amyloid tracer) uptake at 6, 9, 12, 15 and 19 months of age. Increased [^18^F]AV‐45 binding was observed in TG mice as early as 9 months of age in the cortex, but not until 19 months in the hippocampus. Increased [^18^F]DPA‐714 appeared after increased amyloid pathology at 12 and 19 months of age in the cortex and hippocampus respectively. There was a positive correlation between TSPO and amyloid imaging at the 19 months suggesting that as pathology progresses, neuroinflammation worsens. In contrast, Brendel *et al*. (2016) found increased neuroinflammation and metabolism in the PS2APP model prior to significant amyloid pathology in a triple tracer study using [^18^F]GE‐180, [^18^F]FDG and [^18^F]Florabetaben. A small but significant 9% increase in [^18^F]GE‐180 uptake was observed in the TG compared to WT mice at 5 months of age, which continued and reached a 25% increase by 16 months of age. A significant increase in [^18^F]Florabetaben was not observed until 13 months of age indicating that a small increase in basal neuroinflammation may precede pathology development, which is exacerbated after significant plaque burden is observed. Interestingly, these authors noticed higher level of [^18^F]FDG uptake in PS2APP vs WT at 5 months which peaked at 13 months but while [^18^F]FDG uptake increased steadily with age in WT this did not happen in the PS2APP mice (Brendel *et al*. 2016). A strong correlation was found between [^18^F]GE‐180 and [^18^F]Florabetaben uptakes, indicating a positive relationship between neuroinflammation and plaque load, which is in line with results found by Serriere *et al*. in the APP_swe_×PS1_Δe9_ model. Lopez‐Picon *et al*. (2018) demonstrated increased [^18^F]GE‐180 uptake in 17–26 month old APP23 TG mice compared to WT mice. However, a stepwise increase in [^11^C]PiB binding with age was seen in TG mice in the hippocampus and frontal cortex, which was not observed with [^18^F]GE‐180, suggesting that in this model [^11^C]PiB was better suited than[^18^F]GE‐180 to monitor disease progression. Nonetheless, it is important to note that [^18^F]GE‐180 uptake at earlier time‐points was not investigated in this study, and considering the small dynamic range of TSPO expression and limited spatial resolution of microPET, subtle inflammatory changes may not have been detectable using this method.

TSPO and amyloid PET has also been used to assess treatment action. Maeda *et al*. (2007) used [^18^F]FEDAA1106 and [^11^C]PiB to assess the effects of anti‐amyloid treatment in the APP23 mouse. Intra‐hippocampal treatment with anti‐Aβ antibody showed decreases in [^11^C]PiB but increases in [^18^F]FEDAA1106 uptake in the TG mice, indicating that the treatment successfully reduced amyloid burden but was associated with increased neuroinflammation. Similarly, Deleye *et al*. (2017) observed significant reductions in [^18^F]AV‐45 following chronic BACE1 inhibitor treatment, however in contrast to Maeda *et al.,* no alterations in TSPO‐PET were observed. More recently, James *et al*. (2017) demonstrated that [^18^F]GE‐180 permitted detection of decreased cortical and hippocampal neuroinflammation in the APP^L/S^ mouse following treatment with LM11A‐31, a clinical AD treatment which attenuates tau phosphorylation, neurite degeneration and microglial activation. These results highlight the critical role of neuroinflammation in AD pathophysiology, and demonstrate the potential of preclinical TSPO‐PET in therapeutic monitoring and future treatment development. It must be noted however that tracers can behave differently in animals than in human, for example Zanotti‐Fregonara *et al*. (2018) have very recently demonstrated that [^18^F]GE‐180 had a V_T_ 20 fold lower than [^11^C]PBR28 in human brain, demonstrating a very poor extraction fraction and slow brain penetration making [^18^F]GE‐180 particularly challenging to work with in human, while [^18^F]GE‐180 was a suitable tracer in animal models.

#### TSPO PET limitations and future biomarkers

As discussed, one of the limitations of TSPO PET imaging was the potential low signal‐to‐noise ratio and high non‐specific binding of [^11^C]‐R‐PK11195 (Dolle *et al*. 2009). This was alleviated by the development of new and improved second/third‐generation TSPO ligands. Another limitation revealed by both pre‐clinical and clinical TSPO PET studies is that the small dynamic range of TSPO changes in AD. The amplitude of the change in TSPO expression observed in AD is moderate and often overlaps with what is observed in normal aging (Varrone and Nordberg 2015). Overall, these findings support the case for systematic genetic screening for the TSPO polymorphism, the use of new more sensitive TSPO tracers as well as for the search of new suitable biomarkers of neuroinflammation and development of new PET tracers to image them.

Many candidates can be identified among the numerous molecules expressed by glial cells during neuroinflammation; the complexity resides however in the multiple characteristics that the ideal candidate must have to be truly useful from a research and translational perspective. Firstly, such biomarker must be over‐expressed by microglia or astrocytes (possibly but not desirably by both) in disease and exhibit no or minimal expression in the healthy brain. The contrary, i.e. high expression in healthy brain and down‐regulation in disease, could be envisaged but presents the potential issue of quantifying small decreases from the basal control signal. Secondly, the over‐expression of this ideal biomarker should be demonstrated to be associated with well‐established cellular neuroinflammatory processes, and if possible with a specific functional glial phenotype. Finally, existing molecules or class of molecules for this biomarker should exist so that they would form the basis for efficient radiotracer development.

Such biomarkers are being explored and few have emerged as potential candidates. Amongst them, the cannabinoid type 2 receptor holds promises, but the first reports using cannabinoid type 2 receptor tracers suggest that both biomarkers and tracers might not be suitable to monitor neuroinflammation *in vivo* (Vandeputte *et al*. 2012; Ahmad *et al*. 2013). Other targets, such as monoamine oxidase, adenosine receptors, P2X7 receptors or metalloprotease are currently being considered and have been recently and comprehensively reviewed by Janssen *et al*. (2016, 2018).

This search for better biomarkers also links with the possibility to use other modalities than PET to try to measure and assess longitudinally and non‐invasively neuroinflammation. Amongst all modalities, MRS appears to be the one with the highest translational potential since other modalities such as optical imaging do not allow truly non‐invasive clinical investigations.

### MRS

Magnetic Resonance Spectroscopy (MRS) is an NMR‐based technique that can be implemented on the same equipment as MRI. However, MRS, rather than imaging protons (^1^H) in water, measures local concentrations of metabolites containing ^1^H at an abundance of > 1 mM, allowing the detection of biochemical changes *in vivo* in compounds such as N‐acetylaspartate (NAA), myo‐Inositol (mI), creatine + phosphocreatine (Cre), choline‐containing compounds (Cho) and scyllo‐Inositol (See Rae (2014) for a comprehensive review of MRS of the brain). MRS has the ability to track biochemical changes during disease progression and identify early biochemical abnormalities prior to symptom manifestation. Abnormalities in these metabolites have been frequently reported in AD. NAA is considered a neuronal marker (Urenjak *et al*. 1993) and therefore decreasing levels are indicative of neuronal dysfunction or death (Bates *et al*. 1996) and hippocampal spectroscopy analysis has revealed significant reductions in NAA levels, in AD patients compared to both control and MCI subjects (Watanabe *et al*. 2010; Foy *et al*. 2011). However, discrimination is less successful between MCI and healthy controls (Foy *et al*. 2011). Lower NAA levels have also been reported in the posterior cingulate (Zimny *et al*. 2011), anterior cingulate (Shinno *et al*. 2007) and neocortex (Huang *et al*. 2001) of AD patients compared to controls. It has also been claimed that MRS can be used to identify increased levels of neuroinflammation. mI has been suggested to be a glial specific marker (Lazeyras *et al*. 1998), based largely on a report by Brand *et al*. (1993) that glial cells in culture, but not neurons, contain high levels of mI. There is a lack of other studies seriously investigating this contention though it is widely quoted. Many studies have shown mI to be significantly increased in many brain areas including the hippocampal, occipital, parietal and posterior cingulate regions of AD patients compared to healthy controls (Huang *et al*. 2001; Kantarci 2007; Watanabe *et al*. 2010; Silveira de Souza *et al*. 2011). Increased mI levels, whether or not an indication of gliosis cannot be directly linked to activation of microglia as there are no studies which have investigated the metabolic profile of microglia. Nevertheless, if there is an MRS‐marker of inflammation mI is the most likely candidate and may be indicative of glial activation and/or inflammation in AD. However, some inconsistencies remain as some studies have not observed any effect on mI levels (Foy *et al*. 2011), hence further investigation into the meaning of increased level and role of mI is warranted. Another issue to consider in interpreting ^1^H MRS data is how metabolite levels are estimated. Often a ratio to creatine is used, but the assumption that creatine is constant under disease conditions is not always correct – for example in TASTPM transgenic mice Forster *et al*. (2012) reported an increase in creatine in older transgenic mice compared to both wild‐type and younger transgenic animals. It is preferable to use tissue water as a concentration reference rather than metabolites such as creatine or NAA (both commonly used references).

#### Clinical MRS in AD

In line with neuroinflammation as a key driver of AD, increased mI/Cr ratio have been consistently reported in different brain regions of AD patients including temporal lobe (Parnetti *et al*. 1997), posterior cingulate (Kantarci 2007; Shinno *et al*. 2007; Shiino *et al*. 2012; Murray *et al*. 2014), hippocampus (Foy *et al*. 2011; Shiino *et al*. 2012) and parietal grey matter (Rose *et al*. 1999). These areas are associated with early AD pathology and support the presence of neuroinflammation in the early stages of AD development. In addition, decreased NAA/Cr levels have been reported in the same regions (Rose *et al*. 1999; Watanabe *et al*. 2010; Foy *et al*. 2011; Murray *et al*. 2014) as well as the frontal lobe of AD patients (Parnetti *et al*. 1997), suggesting a link between increased neuroinflammation and decrease in neuron viability. Moreover, decreased NAA has been shown to correlate with some specific cognitive tests, including delayed word recall of a learned list and delayed praxis (Foy *et al*. 2011). These results are in line with the progressive pathology and neuronal deterioration seen in AD. Similar results have been reported in MCI patients, with increased mI/Cr and decreased NAA/Cr levels reported in the hippocampus and cingulate (Kantarci 2007; Shiino *et al*. 2012; Targosz‐Gajniak *et al*. 2013). This is in agreement with the anatomical localisation of the brain regions affected early in AD and reinforces the theory that increased early neuroinflammation and decreased neuronal function lead to AD manifestation. However, mI/Cr levels have been shown to predict a progression to AD with a 70% sensitivity and 85% specificity (Targosz‐Gajniak *et al*. 2013) and discriminate between amnesic and non‐amnesic MCI (Kantarci *et al*. 2008). In contrast, Foy *et al*. (2011) investigated hippocampal metabolite concentrations in healthy controls, mild AD and MCI subjects. Significantly lower levels of NAA were seen in AD patients compared to control subjects and MCI subjects. Although there was a trend indicating a difference between NAA levels in MCI and normal subjects, this was not significant. On the other hand, Murray *et al*. (2014) found that increased mI/Cr levels in the posterior cingulate of AD patients were not associated with microglia but were positively associated with Aβ burden, whereas decreased NAA/Cr levels were negatively associated with burden, suggesting mI as a marker of Aβ rather than neuroinflammation. Overall these studies clearly indicate that further investigation is needed to determine the biological relevance of mI signal in AD and other brain conditions.

#### Pre‐clinical MRS in AD

MRS has been carried out in various animal models of AD with results reflecting the clinical situation. Decreased hippocampal and cortical levels of NAA and increased levels of mI have been shown in various animal models of AD (Marjanska *et al*. 2005; Jack *et al*. 2007; Oberg *et al*. 2008; Chen *et al*. 2012). However, results are less consistent in terms of metabolite change and age of alterations than in clinical AD. This overall might be simply due to differences between models in the age of onset and progression rate of AD pathology.

Here, mI has been shown to be significantly increased in TGs when compared to WTs (Yang *et al*. 2011; Forster *et al*. 2013). Yang *et al*. (2011) found that increased mI levels in hippocampal and cortical regions of TG_4510_ mice were supported by increased GFAP and Iba1 staining, demonstrating up‐regulated astrocyte and microglia activation, hence supporting the link between increased mI levels and gliosis. Decreased NAA and Glu ratios were also observed but were not statistically significant. Forster *et al*. (2013) also showed an increase in mI levels in TASTPM mice and demonstrated a significant negative correlation between cognitive function, as assessed by Y‐maze, and mI levels. Significantly decreased NAA levels with age and increased Cr levels at early time‐points (3–9 months) were also reported in this study (Forster *et al*. 2013). In a previous study, Forster *et al*. (2012) carried out NMR *in vitro* using ^1^H‐NMR and did not find significant changes in NAA levels between TASTPM and WT mice. However, they did find increased levels of mI in TG mice at all age except 3 months. This effect was independent of age and indicates that it is a persistent and early characteristic of this model, which however remains to be confirmed in other AD models. They also found increased level of Cr with age, which highlight the potential difficulties of normalising all metabolites to Cr levels (Forster *et al*. 2012). However, this study used whole brain extractions, which may have masked regional effects and may explain why no significant differences were observed in NAA. On the other hand it also emphasizes that the extent of change observed in mI affected the whole brain rather than potentially be region specific. Behavioural testing demonstrates a significant negative correlation between cognitive function as assessed by Y‐maze score and mI levels in TG mice.

Similar to the human case, mI has been suggested to be an early indicator of AD‐like pathology in AD animal models. A study by Chen *et al*. (2009) revealed significant increases in mI levels in APP_swe_×PS1_Δe9_ mice compared to WTs as early as 3 months of age. Moreover, this mI increase occurs prior to plaque development (Garcia‐Alloza *et al*. 2006) or cognitive decline in this model and before the NAA decrease was observed in this study. Similarly, Oberg *et al*. (2008) found increased hippocampal mI levels in an APP_swe_xPS1_PS1M146L_ model of AD as early as 2.5 months. In line with the study by Chen *et al*. (2009), this mI alteration preceded NAA and glutamate (Glu) reductions which appeared at 6.5 months of age. Therefore, mI abnormalities seem to manifest prior to major symptom onset or neuronal damage, indicating neuroinflammation as a crucial player in the early events of AD. In a subsequent study Chen *et al*. (2012) replicated these results and a decrease in Glu/Cr levels at 5 months was also observed. However, it is important to note that other studies did not report significant NAA/Cr reduction in this model until later ages. Jansen *et al*. (2013) reported decreased NAA/Cr levels at only 12 months of age, with no alterations in mI or any other metabolite evident. Similarly, Xu *et al*. (2010) also found decreased NAA/Cr levels to be the only metabolite alteration observed in this model. This effect emerged at only 16 months of age and was associated with hippocampal CA3 pathology. Marjanska *et al*. (2005) observed decreased NAA/Cr levels at 16 months in the same model, as well as decreased Glu/Cr and increased mI/Cr at that age. Similarly, we recently demonstrated decreases in NAA driven by age and genotype in APP_swe_×PS1_Δe9_ mice vs. WT, but did not observe increased mI levels in these mice despite observing increased hippocampal and cortical [^18^F]DPA‐714 uptake. We also reported reduced levels of Glutamate (−53% average across groups from 6 to 18 months) and increased levels of total Choline (+71% average across groups from 6 to 18 months) with age; however those changes seem to be only related to the effect of normal aging (Chaney *et al*. 2018). Other studies have also reported no changes in mI levels between TG and WT mice in other AD mouse models. Dedeoglu *et al*. (2004) found significantly decreased NAA, Glu and glutathione (major antioxidant in the brain) in the frontal cortex of TG_2576_ mice but did not report increased mI levels, however they showed increase in taurine (Taur). It has been suggested that Taur in rodents acts similar to mI in humans and that may account for lack of effect on mI in some mouse studies. It is also possible that the neuroinflammation detected by mI can change with disease progression. Recently, an MRS study was conducted using a new transgenic rat model (McGill‐R‐Thy1‐APP rats) (Nilsen *et al*. 2012). Decreased mI levels were found in the dorsal hippocampus at 3 months compared to WT rats. However, by 9 months of age, mI levels were significantly increased, resulting in higher levels of mI than WT at this age. Yet, by 12 months, no differences in mI levels were identified in the TG rats compared to the WT, indicating that neuroinflammation response may be more complex than originally thought and neuroinflammatory status may change prior to and during disease manifestation and progression. Overall, the large discrepancies between studies regarding the mI levels despite the presence of other *in vivo* or *ex vivo* biomarkers of neuroinflammation leave the question open about the true meaning of elevated levels of mI as biomarker of neuroinflammation. In that line, the study by Pardon *et al*. (2016), in which LPS injection was performed in both WT and APP_swe_×PS1_Δe9_ mice, showed that LPS injection induced no significant changes in mI levels in WT while *ex vivo* examination of the brain showed clear indication of neuroinflammation, and conversely in APP_swe_×PS1_Δe9_ mice there was a strong mI response at 1 and 4 h post‐LPS. On the other hand, LPS injection induced a significant increase in lipid (ML9) and macromolecules levels in WT but not in APP_swe_×PS1_Δe9_ mice. The authors concluded that their results suggested that mI might not be a marker of glial activation and lipid (ML9) and macromolecules levels may be suitable biomarkers of activation of healthy microglia. It is however noteworthy that the amplitudes of mI changes observed with MRS were of small amplitude (4–5%).

In conclusion, more longitudinal studies, using MRS and correlating outcomes to *ex vivo* measurements such as glial activation, amyloid concentration and neuronal integrity markers at each disease stage are needed in order to validate MRS results at a cellular level and elucidate the correlation between mI and neuroinflammatory responses to determine the true biological meaning of mI levels and its suitability as biomarker of neuroinflammation. As with preclinical PET imaging, preclinical MRS is limited in what it can achieve in term of spatial resolution due to the small size of the mouse brains, and once again model of AD in larger species are desirable to realise the full potential of *in vivo* longitudinal imaging studies.

## Conclusions

It has become apparent that AD is a complex multifactorial disease with many contributing factors. In recent years increasing evidence suggests that inflammation has a significant role in AD pathogenesis, however, little is known about the exact contribution. genome wide association studies have also revealed that a number of genes associated with immunity, including TREM2, and CR1, are significantly associated with risk of AD. Altogether these links between neurodegeneration and inflammation suggests that NI may have a crucial role in disease manifestation and/or progression. However, whether microglial activation in AD is harmful or beneficial and whether there is a shift from one to the other with disease progression is still a matter of debate. Hence, further investigation into the complex inflammatory mechanisms in AD is warranted. Recent advances in PET and MRS imaging technology can help to investigate these questions in preclinical and most importantly in clinical settings.

TSPO represents a viable target to image neuroinflammation in AD and is a well validated marker of microglial activation and proliferation in the brain. Thus far, a majority of reports using TSPO PET have shown increased binding in AD patients. However, other studies have demonstrated inconsistencies and similar problems have been reported with MCI. Despite these discrepancies, identification of increased neuroinflammatory responses in MCI and AD patients, as well as rodent models of disease, support the role of inflammation in cognitive dysfunction and implicates it in multiple stages of dementia development.

Although [^11^C]‐R‐PK11195 has been historically the mostly widely used clinical TSPO PET tracer, new tracers seem to offer better sensitivity to detect subtle changes in NI despite facing the challenge of differential affinities due the rs6971 polymorphism. However, one must acknowledge that (i) microglial activation is far more complex than a switch to a phagocytic phenotype and (ii) TSPO as a single PET biomarker for microglial activation is far too limiting and does not encompass the large spectrum of processes involved in neuroinflammation. Therefore, new biomarkers, if possible functionally related to inflammatory processes and/or cell‐specific, are imperative to enable researchers to non‐invasively and quantitatively characterize neuroinflammation in AD.

Finally, it is important to note that microglial activation is not specific to AD and appears in many neurodegenerative disorders. As increased microglial activation is seen in MCI, this suggests that neuroinflammation is a key factor in the early stages of AD development and could therefore be a target for therapeutic intervention. Moreover, NI can also be used as a biomarker for monitoring the efficacy of immune‐modifying therapy in conjunction with other specific biomarker such as [^11^C]PIB or other tracers for PET amyloid imaging and cognitive and psychological testing, although considering the cost of PET this would probably be limited to investigational studies or clinical trials rather than routine screening. However, in recent years, advances have been made in our understanding of microglia and astrocytes biology with their origins being redefined and functions being reviewed (Gomez‐Nicola and Perry 2015; Sarlus and Heneka 2017; Ising and Heneka 2018). We now know that glial cells are immensely dynamic cells that adapt rapidly to their micro‐environment but much regarding their functions remains to be resolved. Therefore, to fully exploit the potential of NI as a therapeutic target, a better understanding of the biology and the time‐course of NI events is still needed. In this perspective, imaging techniques are going to be essential to investigate NI processes non‐invasively in patients and in animal models and pave the way for future anti‐inflammatory therapies in neurodegenerative disease.
